# Association mapping reveals novel serpentine adaptation gene clusters in a population of symbiotic *Mesorhizobium*

**DOI:** 10.1038/ismej.2016.88

**Published:** 2016-07-15

**Authors:** Stephanie S Porter, Peter L Chang, Christopher A Conow, Joseph P Dunham, Maren L Friesen

**Affiliations:** 1 School of Biological Sciences, Washington State University, Vancouver, WA, USA; 2 Section of Molecular and Computational Biology, Department of Biological Sciences, University of Southern California, Los Angeles, CA, USA; 3 Department of Plant Pathology and Nematology, University of California, Davis, CA, USA; 4 Department of Plant Biology, Michigan State University, East Lansing, MI, USA

## Abstract

The genetic variants that underlie microbial environmental adaptation are key components of models of microbial diversification. Characterizing adaptive variants and the pangenomic context in which they evolve remains a frontier in understanding how microbial diversity is generated. The genomics of rhizobium adaptation to contrasting soil environments is ecologically and agriculturally important because these bacteria are responsible for half of all current biologically fixed nitrogen, yet they live the majority of their lives in soil. Our study uses whole-genome sequencing to describe the pan-genome of a focal clade of wild mesorhizobia that show contrasting levels of nickel adaptation despite high relatedness (99.8% identity at 16S). We observe ecotypic specialization within an otherwise genomically cohesive population, rather than finding distinct specialized bacterial lineages in contrasting soil types. This finding supports recent reports that heterogeneous environments impose selection that maintains differentiation only at a small fraction of the genome. Our work further uses a genome-wide association study to propose candidate genes for nickel adaptation. Several candidates show homology to genetic systems involved in nickel tolerance and one cluster of candidates correlates perfectly with soil origin, which validates our approach of ascribing genomic variation to adaptive divergence.

## Introduction

A central challenge in microbial evolutionary ecology is identifying genomic variation responsible for environmental adaptation. Local adaptation results when environment-specific selection is stronger than migration and builds local reservoirs of genetic diversity within a population's biogeographic structure (Martiny *et al.*, 2006; Reno *et al.*, 2009; Hanson *et al.*, 2012; Cordero *et al.*, 2012b; Cordero and Polz, 2014; Shapiro and Polz, 2014). Current models of microbial diversification require selection for habitat-specific adaptive variants leading to the formation of stable genomic clusters, that is, ‘ecotypes' (Konstantinidis and Tiedje, 2005; reviewed in Polz *et al.*, 2013; Shapiro and Polz, 2014). In genome-wide sweeps, environmental selection causes clonal expansion of the first genome that acquires an adaptive variant (Cohan, 2002; Shapiro and Polz, 2014). Sweeps initially lower diversity, which is replenished by subsequent mutations (Shapiro and Polz, 2014). In contrast, gene-specific sweeps occur when recombination across habitats erodes genome-wide linkage with environment-specific adaptive variants (Cordero *et al.*, 2012b; Shapiro *et al.*, 2012; Polz *et al.*, 2013; Shapiro and Polz, 2014; Rosen *et al.*, 2015). Empirical genomic data supporting these different paths to microbial ecotypic diversification are relatively sparse (Polz *et al.*, 2013; Cordero and Polz, 2014; Shapiro and Polz, 2014). Thus, characterizing adaptive variants and their genomic context remains a key frontier (Shapiro *et al.*, 2009; Zaneveld *et al.*, 2011).

A transformative insight from high-throughput bacterial population genome sequencing (Stapley *et al.*, 2010; Zaneveld *et al.*, 2011) is that closely related strains share a core genome but that each has a suite of varying accessory (flexible) genes (Reno *et al.*, 2009; Polz *et al.*, 2013); collectively these make up the pan-genome (Kettler *et al.*, 2007; Lapierre and Gogarten, 2009; Reno *et al.*, 2009; Denef *et al.*, 2010a). If genome-wide linkage is low, variation in the core and accessory genomes may have distinct biogeographical patterns. For example, the core genomes of *Rhizobium leguminosarum* (Kumar *et al.*, 2015) and *Vibrio cholerae* (Boucher *et al.*, 2011) are globally well-mixed, while the accessory genome is ecologically differentiated. The core genome encodes basic functions necessary for the common niche of the sampled isolates (Lapierre and Gogarten, 2009; Polz *et al.*, 2013). Adaptation via the core genome could be constrained by slow rates of nucleotide substitution or genome-wide selective sweeps (Gevers *et al.*, 2005; Cohan and Perry, 2007). However, adaptive alleles within the core genome demonstrate that recombination can act rapidly enough for gene-level sweeps to occur in response to temperature and latitude (terrestrial fungus *Neospora*; Ellison *et al.* (2011)), and substrate particle size (marine *Vibrio* bacteria; Shapiro *et al.* (2012)). The accessory genome, with its shifting complement of genes that can be lost or gained via horizontal transfer (Polz *et al.*, 2013), is considered the primary reservoir of microbial adaptive variation, as well as containing transient, non-adaptive genes (Kuo and Ochman, 2009; Lapierre and Gogarten, 2009; Andam and Gogarten, 2011; Smillie *et al.*, 2011). Adaptive accessory genes have been identified for phosphorus availability (marine *Prochlorococcus* and *Pelagibacter*; Coleman and Chisholm (2010)) and successional context (acid mine drainage *Leptospirillum* bacteria; Denef *et al.* (2010a)). An environment-specific horizontally transmitted accessory gene pool can connect otherwise unrelated strains (reviewed in Polz *et al.*, 2013).

Recent work presented a collection of locally adapted wild *Mesorhizobium* strains across naturally nickel-enriched serpentine soils paired with nearby low-nickel soils (Porter and Rice, 2013). Rhizobia are an accessible population because they disperse through soil between generations of symbiotic association with their host plant (here, *Acmispon wrangelianus*) and are readily culturable in the laboratory (Sprent, 2007; Oldroyd *et al.*, 2009). Heavy metal enrichment and unusual ionic ratios in both natural serpentine soils (Brady *et al.*, 2005; Turner *et al.*, 2010) and anthropogenic mine spoils (McNeilly, 1968) can drive local adaptation in plants. While rhizobia are often sensitive to heavy metals (Giller *et al.*, 2009), these *Mesorhizobium* exhibit local adaptation to nickel in culture: strains originating from high-nickel serpentine soil had higher fitness under high-nickel levels than strains originating from low-nickel non-serpentine soil, and strains originating from non-serpentine soil had higher fitness in low-nickel media than strains from serpentine (Porter and Rice, 2013).

We use whole-genome sequencing to describe the pan-genome of a focal clade of mesorhizobia that show contrasting levels of nickel adaptation. We describe how spatial distance and soil type structure the core and accessory genomes. Using a genome-wide association study, we identify genomic variants that are statistically associated with strain growth in nickel-containing medium and compare these with known heavy metal resistance mechanisms (Sa-Pereira *et al.*, 2009; Mengoni *et al.*, 2010). We ask whether adaptive variants (i) reside in the core or accessory genome, (ii) are clustered and (iii) are shared with distantly related lineages.

## Materials and methods

**Figure 1:**
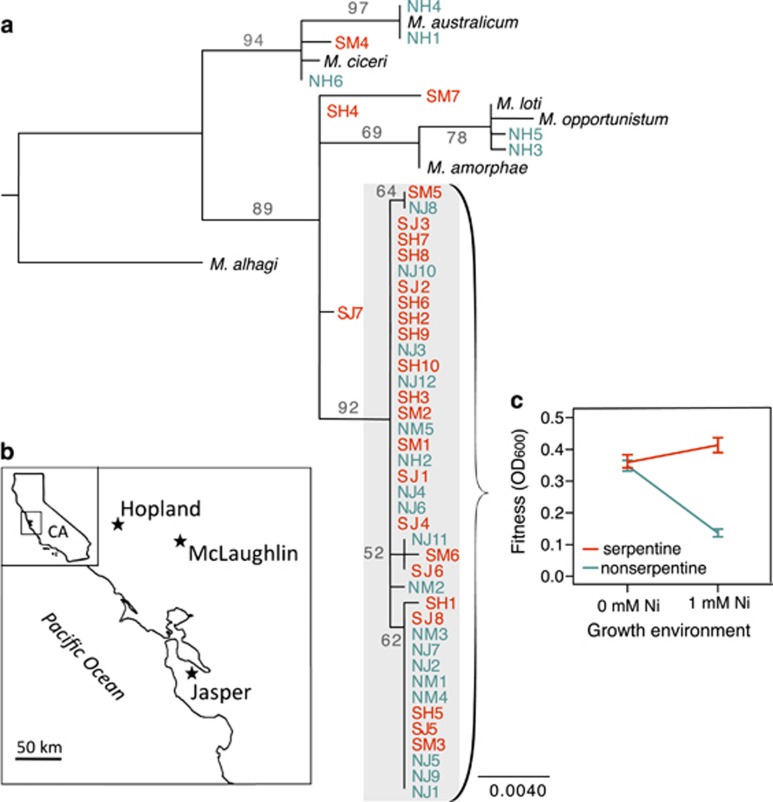
Differential nickel adaptation in wild *Mesorhizobium.* (**a**) Phylogenetic tree based on 16S Sanger sequence data, with bootstrap values (teal: serpentine; red: non-serpentine soil of origin; gray background: focal clade). (**b**) Collection sites in California (CA) (star: a reserve where adjacent serpentine and non-serpentine soils were sampled). (**c**) Growth data for strains from the focal clade in the presence and absence of nickel in liquid media (OD_600_: optical density at 600 nm).

*Mesorhizobium* strains with varying responses to nickel were isolated from field-collected *A. wrangelianus* root nodules by Porter and Rice (2013) (Figure 1) from serpentine soil, which is naturally enriched in toxic levels of nickel (3.1–86.5 mg Ni kg^−1^), and from spatially adjacent non-serpentine soils containing lower levels of nickel (0.8–6.85 mg Ni kg^−1^) at each of three Californian reserves (Figure 1). We selected 38 strains from a focal 16S subclade (99.8% identity over 1340 bp) present in both soil types and all three reserves that exhibit adaptation to soil nickel, as well as 9 strains that captured the diversity of sympatric *Mesorhizobium* clades (Figure 1a). Within the focal clade, serpentine strain fitness is higher than non-serpentine strain fitness under high nickel (F_1,34_=78.4, *P*<0.0001; Figure 1c), while serpentine and non-serpentine strain fitness is similar under low nickel (Figure 1). Supplementary Information 1 contains additional details regarding methods.

DNA extraction and genomic library construction followed Dunham and Friesen (2013). Libraries were sequenced in a paired 76 bp format on an Illumina GAIIx (San Diega, CA, USA). Draft genomes were assembled with the A5 pipeline (Tritt *et al.*, 2012). Genes were annotated using a two-stage procedure. First, draft genomes were searched for ‘reference' genes homologous to 7272 protein-coding sequences in the complete *Mesorhizobium huakuii bv. loti* MAFF303099 genome (BLASTX alignment with >80% overlap, *E-*value <1E-20, and protein sequence identity >50%). Next, we identified ‘*de novo*' genes in the wild strains that were absent in *M. huakuii bv. loti*. Regions of the draft assemblies that did not contain reference genes were aligned using BLASTX to the non-redundant GenBank CDS translations+PDB+Swiss-Prot+PIR+PRF (nr) database using the same BLASTX parameters. Orthology among *de novo* genes was determined using reciprocal BLAST (Supplementary Information 1). Annotation was conducted with Blast2GO v.2.3.5 (Conesa *et al.*, 2005) and UniProt (UniProt Consortium, 2015).

‘Core' genes are present in all strains for a given subset (focal clade: 38 strains; full wild set: 47 strains; full set with references: 53 strains). ‘Accessory' genes are absent from one or more strain in each subset. Delineation of core and accessory was calculated for each of the three subsets. For genes in the core genome across all 53 strains, we performed multiple sequence alignments to identify single-nucleotide polymorphisms (SNPs) (ClustalW; Larkin *et al.*, 2007). Simulation verified the efficacy of our methods in identifying accessory genes (Supplementary Figure S1).

To compare core and accessory genomes, we calculated GO enrichment tests (R Bioconductor package ‘GOstats' Falcon and Gentleman (2007)) and rates of non-synonymous (Ka) to synonymous (Ks) substitution (KaKs Calculator; Zhang *et al.*, 2006). Strain relatedness was assessed using core genome allelic variation and accessory gene presence/absence. We used a population-based perspective allowing horizontal gene transfer implemented by NeighborNet (SplitsTree v4.12.8; Huson, 1998, 2005).

To analyze biogeographic patterns we used ‘adonis' in the R package ‘vegan' (Dixon, 2003) with bootstrapping. To infer population structure and probabilistically assign individuals to populations, we ran STRUCTURE v2.3.2.1 (Pritchard *et al.*, 2000) on each of 10 replicate SNP subsets sampled every 25 SNPs. We used delta K with K ranging from 1 to 8 (Evanno *et al.*, 2005).

We conducted genome-wide association mapping (Friesen and von Wettberg, 2010) of growth in the presence of nickel for (i) gene content in the accessory genome, and (ii) SNPs in the core genome, excluding singletons and regressing out STRUCTURE effects (see Supplementary Information 1). Significance of the Wilcoxon test was assessed using Bonferroni correction and false discovery rates (FDR) (Hochberg and Benjamini, 1990). Candidates were further annotated using TrEMBL and Swiss-Prot. We examined draft genome contigs containing FDR<0.10 candidates to assess clustering of candidate genes. A cluster was defined as a region on a single contig with at least four tandem candidate genes, and expanded to include nearby candidate genes until no candidate gene was found for 10 kb on either end.

We tested whether candidate accessory genes are shared with lineages that are more phylogenetically distant from the focal clade than expected by chance. For each accessory gene, we identified the lineage bearing the closest homolog in Genbank and calculated the distance between this bacteria and the focal clade at 16S. We compared the distribution of distances for candidate vs non-candidate accessory genes using a KS test.

## Results

### Population pan-genomics

We characterized the pan-genome of 47 strains of wild symbiotic *Mesorhizobium* isolates collected across replicated serpentine and non-serpentine Californian soils (Figure 1). Draft genomes ranged in size from 5.75 to 7.79 Mbp (median 6.61 Mbp; Supplementary Table S1). Assemblies used from 95% to 98% of filtered reads for a median coverage of 70 × (42–130 ×). The number of genes identified in each draft genome ranged between 5454 and 6725, with a median of 6078. Draft genomes had a median of 56.5 contigs (21–273) and median N50 of 358 kbp (129–911 kbp).

**Figure 2:**
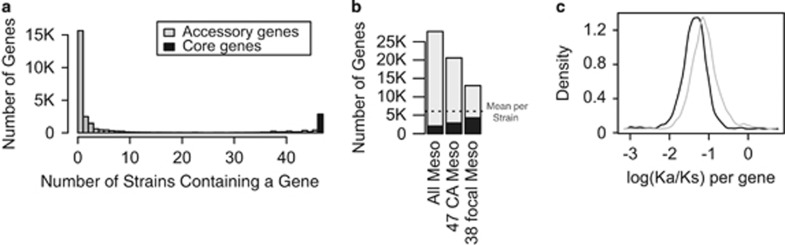
Core and accessory genome compartments of wild *Mesorhizobium* are distinct. (**a**) Distribution of genes across strains. (**b**) Pan-genome size for different groups of strains. (**c**) Distribution of non-synonymous to synonymous substitution ratio (log10 Ka/Ks) for core and accessory genes.

Across our 47 wild strains, genes tended to be rare or ubiquitous (Figure 2a). Across our 47 wild strains and 6 fully sequenced relatives, 27 802 genes are present in at least one strain with 2059 core genes in all 53 strains (Figure 2b). Wild Californian strains have a pan-genome containing 20 582 genes and a core genome of 2874 genes, while our focal clade has a pan-genome of 13 049 genes and a core genome of 4308 genes (Figure 2b). Within the core genome of the complete set of 53 strains, there were 1 106  970 segregating SNPs called in all strains; 212 192 of these varied within the focal clade and were used for phenotype associations.

Core and accessory genomes exhibit contrasting patterns of functional enrichment and molecular evolution. Compared with the core genome, accessory genes show the strongest enrichment for the molecular function, nucleotide binding (GO:0003676: all strains *P=*4.42E-10; wild strains *P=*8.51E-13), and the biological process, DNA metabolism (GO:0006259: all strains *P=*1.45E-36; wild strains *P=*2.36E-49). The accessory genome is also strongly enriched in DNA recombination (GO:0006310: all strains *P=*1.37E-35; wild strains *P=*2.69E-50), with all but one gene annotated with this term located in the accessory genome (all strains 528/529 genes; wild strains 430/431 genes). Finally, the accessory genome is enriched in cellular compartment terms related to the cell membrane, including membrane (GO:0016020: all strains *P=*1.42E-23; wild strains *P=*6.08E-20), intrinsic to membrane (GO:0031224: all strains *P=*7.43E-10; wild strains *P =*1.64E-05) and membrane part (GO:0044425: all strains *P=*8.33E-09; wild strains *P=*5.24E-05) (Supplementary Table S2).

Compared with the core genome, the accessory genome showed elevated rates of protein sequence evolution relative to genome sequence change, reflected in the ratio of non-synonymous to synonymous substitutions (Wilcoxon rank sum test on log10 values: *W*=4 337 428, *P*<2.2E-16; Figure 2c).

**Figure 3:**
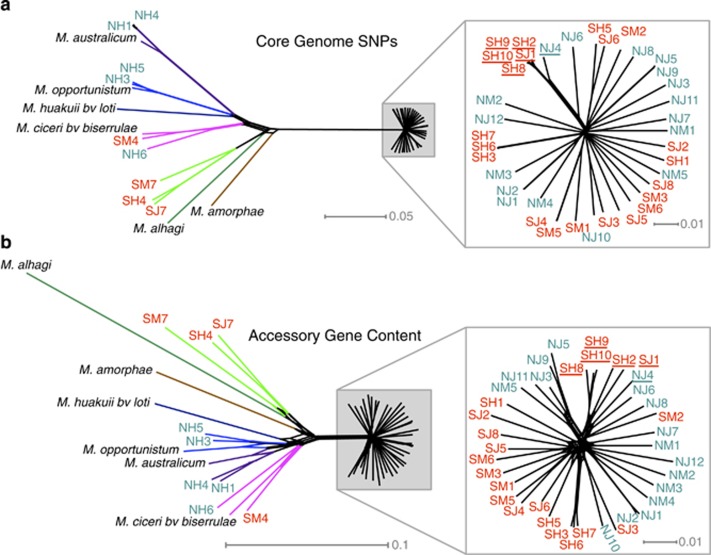
NeighborNet diagrams of strain relatedness indicate the focal clade is genomically cohesive. (**a**) Relatedness based on SNPs in the core genome; scale bar indicates SNP divergence, scaled by the number of SNPs per bp of aligned core genome. (**b**) Relatedness based on variation in accessory gene content; scale bar indicates divergence of accessory gene presence/absence. Zoomed region depicts the focal clade (teal: serpentine soil origin; red: non-serpentine soil origin). Strains fully assigned to the minority STRUCTURE subgroup within the focal clade are underlined. Non-focal clades are color-coded for ease of comparison.

Using NeighborNet, the core genome of the focal clade of 38 strains is divergent from six fully sequenced *Mesorhizobium* strains as well as the remaining 9 wild strains (Figure 3). NeighborNet trees show more reticulation in the gene content network than the SNP network (Figure 3). However, SNPs in the core genome and variation in the accessory genome show congruent topologies (Figure 3). Furthermore, both core and accessory genome variation show that the focal clade subnet is approximately a star with little deep structure and no strong signal of soil type or reserve (Figure 3).

STRUCTURE and the delta K method demarcate two clusters within the 47 wild strains, with the focal clade distinct from the more distantly related strains. Within the focal clade, there is weak genomic structuring among collection locations and soil types. STRUCTURE analysis revealed two subgroups within the focal clade core genome (Supplementary Figure S2 and Supplementary Table S3). Divergence within the focal clade is not split cleanly by soil type or geographic separation: averaged over multiple STRUCTURE runs, the minority STRUCTURE subgroup contains five fully assigned strains from serpentine soil from Jasper Ridge and Hopland and one 92.8% assigned strain from non-serpentine soil from Jasper Ridge. Other strains contained 0.011–38.0% of the minority cluster (Supplementary Figure S2). Nonparametric analysis of variance revealed that a significant but relatively small amount of genetic variation is explained by reserve (a proxy for geographic distance) and soil type (Supplementary Figure S3). This analysis also revealed that soil type explains more of the variance in the accessory genome than in the core genome, whereas reserve does not (see 95% confidence intervals; Supplementary Figure S3).

### Adaptive variants

**Figure 4:**
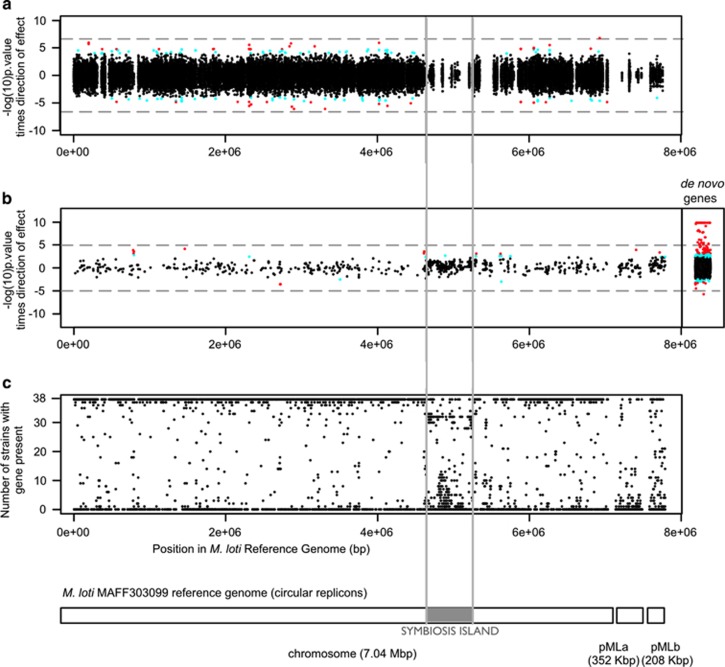
Loci associated with nickel adaptation do not cluster positionally relative to the reference *Mesorhizobium huakuii bv. loti* genome. Significance of genome-wide association study association of genes with nickel tolerance, corrected for weak genetic structure, in (**a**) the core genome and (**b**) the accessory genome (black points: genes not significant; cyan points: 10% FDR significant; red points: 5% FDR significant; dashed gray line, Bonferroni significance level; *de novo* genes are present in the wild strains but absent in the *M. huakuii bv. loti* reference). (**c**) Prevalence of each reference *M. huakuii bv. loti* gene across the wild focal population, shown by the number of wild strains containing each gene (solid gray line: location of the symbiosis island in the reference *M. huakuii bv. loti* genome). Physical positions of loci in (a–c) correspond to the positions of homologous regions in the *M. huakuii bv. loti* reference genome diagram; *de novo* genes in (b) are plotted with jitter because positional information is lacking for them.

The presence or absence of 202 accessory genes was associated with a strain's growth phenotype in nickel-enriched media at the 10% FDR significance level (Figure 4 and Supplementary Table S4; 33 genes significant after Bonferroni correction). In contrast, there were no accessory genes associated with growth in the low-nickel media, even at the 20% FDR level.

At the stringent Bonferroni-corrected threshold, several nickel-associated accessory genes are predicted to have functional roles in metal tolerance according to homology-based annotation (Table 1, bolded; Table 2). Candidates that share homology with proteins with functional validation in the literature include a putative high-affinity nickel transporter, a chromate ion transporter, a metal cation efflux system protein, a manganese and iron superoxide dismutase protein, and an opine dehydrogenase (Table 2). At 10% FDR significance, four additional candidate accessory genes have compelling annotations: a cobalt–zinc–cadmium resistance protein, a nickel import ATP-binding protein, an ABC-type dipeptide/oligopeptide/nickel transport system and a nickel–cobalt–cadmium resistance protein (Table 2).

In the core genome, 199 biallelic SNPs associate with growth in nickel-enriched media at 10% FDR significance (Table 1, Figure 4 and Supplementary Table S5). The single Bonferroni-significant SNP is located in a gene annotated as succinoglycan transport protein exoP (Table 2). No SNP candidates reside in genes homologous to proteins with functions related to nickel.

**Figure 5:**
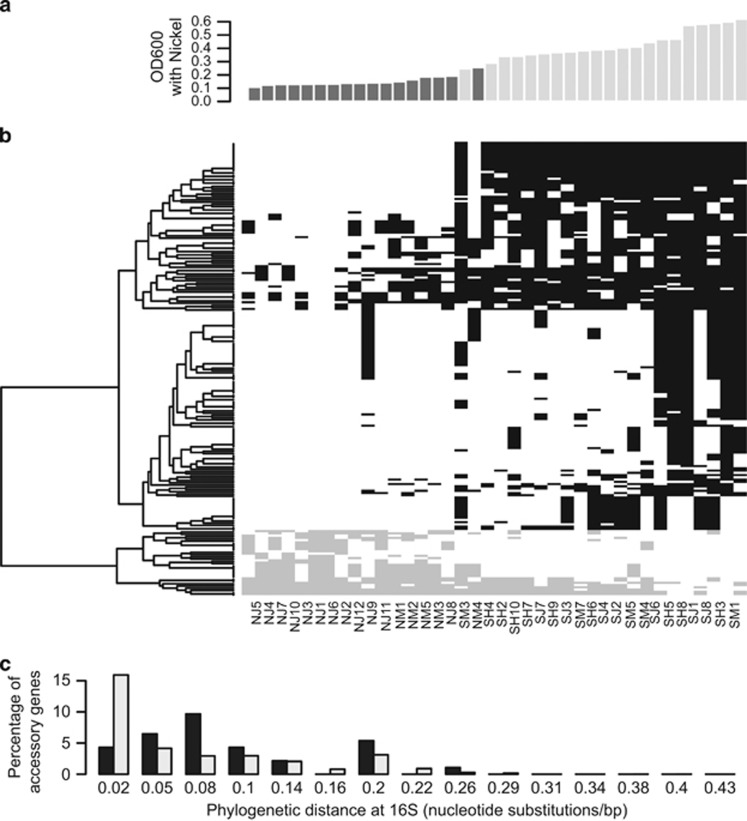
Candidate loci from the accessory genome do not strictly co-segregate and tend to be shared with lineages that are relatively distantly related to *Mesorhizobium*. (**a**) Growth of each focal population strain in media containing 1 mm nickel (dark gray: non-serpentine origin; light gray: serpentine origin). Strain identity is indicated in corresponding column of (**b**). (**b**) Pattern of co-segregation of accessory gene nickel adaptation candidates (10% FDR set) across the 38 strains in the focal population (black: candidates associated with increased growth in nickel; gray: candidates associated with decreased growth in nickel). Genes are ordered based on co-segregation as indicated in the dendrogram. (**c**) Phylogenetic distance at 16S between focal clade mesorhizobia and the bacterial lineage with the closest homolog to each accessory gene (dark gray: FDR 1% accessory gene candidates; light gray: all accessory genes with a match for which we could extract 16S information).

**Figure 6:**
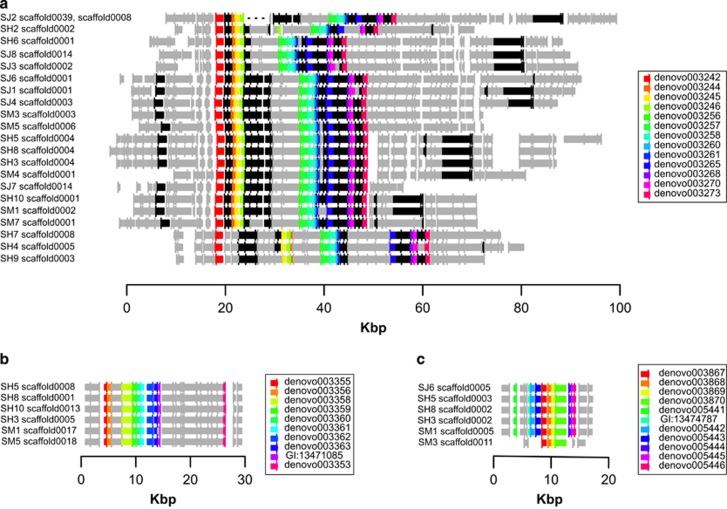
Clusters of candidate nickel adaptation genes. (**a**) Cluster A, found in all serpentine-origin strains, contains 32 Bonferroni-significant genes (black), 13 of which are only found in serpentine strains (rainbow). Other genes are gray. (**b**) Cluster B contains 10 genes significant at FDR 10% (rainbow); other genes in gray. (**c**) Cluster C contains 11 genes significant at FDR 10% (rainbow); other genes in gray. Flat arrowheads denote the 3′ end of genes.

Candidates from the accessory and core genomes do not fully co-segregate across strains (Figure 5). We identified seven clusters with at least 10 candidate genes, with 3 of these occurring only in serpentine-origin strains. These three clusters span a total of ~131 Kbp and contain 97 of the 202 candidates with 10% FDR significance. The largest cluster (Cluster A) spans 86 Kbp and contains 77 candidate genes, including 32/33 Bonferroni-significant candidates, all 13 of the candidates that assort perfectly by soil type, and all candidates with supporting evidence for metal tolerance in the literature (Table 2), except for GI:13475426 (Supplementary Table S6). We determined that the 13 candidates that assort perfectly by soil type are within an ~30 Kbp region in Cluster A (Figure 6a). However, their gene order and contig position is variable among strains due to non-candidate gene indels, local duplications and transversions (Figure 6a). Two other clusters span a region of 30 Kbp (Cluster B) and 15 Kbp (Cluster C) and include 10 and 11 candidate genes, respectively (Figures 6b and c). Clusters B and C are always found on different contigs, and each is carried by six serpentine strains, with four strains containing both clusters. Clusters B and C have conserved gene content and order, and each contains genes primarily from the de novo assembly, with only one reference gene each; many of the genes in these clusters are annotated as conjugal transfer proteins or transposases (Supplementary Table S6). Groups of reference genes, within and flanking the ends of each cluster, were not consistently co-located in the *M. huakuii bv. loti* reference genome. This could be due to structural divergence between the focal population and the reference, or to a complex process of integration of horizontally acquired material into the genome, and precludes the identification of putative cluster insertion sites.

In the focal clade, nickel-adaptation candidates from the accessory genome are shared with lineages that are more phylogenetically distant to the focal clade than expected. This pattern was strongest for nickel-adaptation candidates at 1% FDR—of these 43 genes, 33 were found in bacterial lineages with existing 16S sequence data. On average, this set was 3.3% more divergent in terms of 16S base pair similarity (0.033 average nucleotide identity) from *M. huakuii bv. loti* than lineages sharing the closest homologs to non-candidate accessory genes (Wilcoxon test *P*=0.023; Figure 5c). This result also held for the 5% FDR set of genes (62/87 genes with 16S distance, Wilcoxon difference 0.006, *P*=0.034) and the pattern was marginally significant for the FDR 10% genes (128/202 genes with 16S distance, Wilcoxon difference 0.0029, *P*=0.076).

## Discussion

Understanding rhizobial adaptation to soil environments is important because symbiotic rhizobia are responsible for half of all current biologically fixed nitrogen (Gruber and Galloway, 2008) yet live the majority of their lives in soil. Genomic analysis of a population of wild *Mesorhizobium* from California shows that adaptive differentiation across contrasting soil nickel levels occurs within an otherwise cohesive genome and gives insight into the biogeography of the pan-genome, the identity of adaptive genomic variants and the process of environmental adaptation in bacteria.

### Genomic context of environmental adaptation

The focal *Mesorhizobium* clade in this study is a genomically cohesive population that is widely dispersed across habitat boundaries yet harbors ecotypic variation for adaptation to high-nickel soils. Phylogenomic analysis of this population (99.8% 16S similarity) yields a star-like topology for both variation in the core and accessory genomes, with relatively long terminal branches that reflect strain-specific mutations similar to those observed in other rhizobial populations (Bailly *et al.*, 2011; Kumar *et al.*, 2015). STRUCTURE analysis further supports this population's distinctness from other sympatric strains. The lack of phylogenetic structure within the focal population is consistent with recombination homogenizing the core and accessory genomes among soil types and across hundreds of kilometers. A similar pangenomic pattern was observed in early stages of habitat-based ecotypic differentiation in a wild population of marine *Vibrio cyclitrophicus* (Shapiro *et al.*, 2012), and *Ensifer meliloti* rhizobia display similar homogenizing levels of recombination across host species and thousands of kilometers (Epstein *et al.*, 2012).

Bacterial pan-genomes typically consist of distinct core and accessory genome compartments and our *Mesorhizobium* draft genome sequences recapitulate this pattern. Our assemblies appear to capture the vast majority of genomic content despite not being fully closed, because they are similar in size to related closed genomes and a high fraction of raw reads are used in the assembly (95–98%). Contrasting patterns of functional enrichment and molecular evolution in core vs accessory *Mesorhizobium* genomic compartments demonstrate that they experience different evolutionary processes. The accessory genome is strongly enriched in genes related to DNA recombination and metabolism, and many appear to reside in the symbiosis island region. These characteristics accord with the concept that horizontally transferred genes occur at sites of recombination (Karberg *et al.*, 2011) and that intra-population gene content variation results largely from phage, plasmid and transposon movement (Denef *et al.*, 2010b). The accessory genome is also enriched for cell membrane cellular compartment GO terms, similar to findings from studies of biotic drivers of rapidly evolving cell-surface molecules, membrane-spanning transporters and cell membrane modifiers, which play key roles in microbial biotic interactions (Kettler *et al.*, 2007; Cordero *et al.*, 2012a; Polz *et al.*, 2013). Biotic selection, environmental selection, or mutational bias could underlie the elevated rates of protein sequence evolution relative to genome sequence change that we observe in the accessory genome; although most core and accessory genomes have a negative log ratio of non-synonymous to synonymous substitution which suggests primarily purifying selection, as observed in *Ensifer* rhizobia (Epstein *et al.*, 2014). Higher network reticulation for the accessory genome compared with the core genome is consistent with a pattern of horizontal exchange of accessory genes among lineages (Polz *et al.*, 2013). However, at broader phylogenetic scales these *Mesorhizobium* core and accessory genomes yield congruent topologies, in contrast to the divergent phylogenetic processes acting at core and accessory genomic compartments in *Rhizobium leguminosarum* (Kumar *et al.*, 2015).

Bacterial populations are often weakly structured by geographic distance, particularly in taxa that can disperse via aerial dust or surface water (Martiny *et al.*, 2006; Hanson *et al.*, 2012). We find that both geographic location and soil type explain low but significant amounts of genomic variation within our focal population. Environmental selection appears to be stronger and play a relatively large role in structuring accessory genes compared with core genes, as accessory genes showed greater divergence across soil types than core genes, but equivalent isolation by distance. Given that only a few migrants per generation are sufficient for complete population admixture, our observation of genetic structure across adjacent serpentine and non-serpentine soils points to strong environmental selection, particularly since gene flow, in the present study and other studies, commonly occurs over geographic distances several orders of magnitude higher (Shapiro and Polz, 2014). Our study adds to a growing list of microbial populations that remain cohesive at neutral loci despite large-scale geographic separation (Shapiro and Polz, 2014).

### Genetic variants underlying environmental adaptation

As phylogenetic and biogeographic evidence indicate that our focal *Mesorhizobium* population experiences ongoing recombination, we undertook a genome-wide association study to detect genetic variants associated with nickel adaptation (Friesen and von Wettberg, 2010; Chen and Shapiro, 2015). The majority of candidate variants for nickel adaptation are gene content variants in the accessory genome rather than allelic variants in the core genome. Numerous candidate genes have high statistical support and homology to loci that have been experimentally demonstrated to be involved in nickel tolerance, consistent with the perspective that the accessory genome is a reservoir of adaptive genes (Coleman and Chisholm, 2010; Polz *et al.*, 2013), though there could be rare genes associated with nickel tolerance we are unable to detect with genome-wide association study.

Nickel efflux systems prevent toxic intracellular nickel concentrations (Macomber and Hausinger, 2011) and are upregulated in response to metal exposure in other mesorhizobia (Maynaud *et al.*, 2013). We find several candidate loci with putative roles in the transport of nickel or other divalent cations—common resistance proteins often underlie adaptation to multiple divalent cations (Macomber and Hausinger, 2011). Candidate ‘denovo000014' is homologous to a high-affinity nickel transporter in *Ensifer medicae* and the nickel/cobalt efflux system RcnA. RcnA is a membrane-bound polypeptide that contributes to nickel resistance (Rodrigue *et al.*, 2005; Koch *et al.*, 2006; Iwig *et al.*, 2006) and nickel homeostasis (Macomber and Hausinger, 2011). Candidate ‘denovo003248' shares homology with a putative chromate transport protein in *E. meliloti* and chromate transport protein chrA1, a plasmid-borne gene that contributes to chromate efflux in metal-tolerant *Cupriavidus metallidurans* (Nies *et al.*, 1990; Juhnke *et al.*, 2002). Elevated soil chromate may be associated with elevated soil nickel concentrations (Brady *et al.*, 2005), or this transporter could recognize a negatively charged nickel complex. Candidates ‘denovo003258' and ‘denovo000013' are related genes that share homology with a probable cobalt–zinc–cadmium efflux system protein in *Rhzobium etli,* as well as cation efflux protein Rv2025c, which encodes a functionally validated cation diffusion facilitator (CDF)-family metal exporter in *Mycobacterium tuberculosis* (Campbell *et al.*, 2007). Rv2025c is de-repressed by Ni^2+^ and Co^2+^, is controlled by the high-affinity Ni^2+^/Co^2+^ sensor KmtR and may use Ni^2+^ as a substrate (Cubillas *et al.*, 2013). Candidate ‘denovo003460' has homology to an inferred ABC-type dipeptide/oligopeptide/nickel transport system in *Methylophaga aminisulfidivorans* (Han *et al.*, 2011). Accessory gene candidate ‘GI:13475426' is annotated as a nickel–cobalt–cadmium resistance protein in *Mesorhizobium huakuii bv. loti* and is homologous to NccN, a nickel–cadmium–cobalt resistance protein that contributes to nickel efflux in *Alcaligenes xylosoxydans* (Schmidt and Schlegel, 1994). Genes in the ncc operon share close homology with those in the cnr operon, a cobalt nickel efflux system integral to nickel tolerance in *Bradyrhizobium* from serpentine soil (Chaintreuil *et al.*, 2007). Candidate ‘denovo003135'is homologous to a putative nickel import ATP-binding protein, NikD, in *Methlyophaga thiooxydans* DMS010 (Han *et al.*, 2011).

Three other candidates are annotated with functions that suggest involvement in symbiosis and/or environmental stress. Candidate ‘denovo003250' is homologous to iron superoxide dismutase protein ChrC, which contributes to nickel tolerance in *C. metallidurans* (Juhnke *et al.*, 2002). Superoxide dismutase scavenges superoxides produced during oxidative damage and also confers nickel tolerance in *Escherichia coli* (Macomber and Hausinger, 2011). Candidate ‘denovo003265' is annotated as an opine dehydrogenase (Asano *et al.*, 1989; Dairi and Asano, 1995). Opines, specialized bacterial metabolites produced during symbiosis, have roles in host colonization and communication (Denison, 2000; Savka *et al.*, 2002). As heavy metals can inhibit symbiosis initiation (Mengoni *et al.*, 2010), opines might play a role in symbiotic adaptation to serpentine soils. Finally, we find a well-supported candidate SNP in the succinoglycan transport protein ExoP. The extracellular polysaccharide succinoglycan is involved in symbiosis (Jones *et al.*, 2008) and can also mediate biofilm formation, which could contribute to nickel tolerance (Harrison *et al.*, 2004); extracellular polysaccharide molecules may also bind metal directly and contribute to tolerance (Gadd, 1990).

### The evolutionary processes underlying environmental adaptation

The focal *Mesorhizobium* population exhibits environment-specific adaptive variants, yet lacks strong genome-wide differentiation between environments, suggesting that gene flow across environments may maintain genomic cohesion within this population. This is consistent with a population classification of ‘stage 2' in Shapiro and Polz's (2014) continuous speciation spectrum model. The best supported adaptive variants are clustered positionally within a 30 Kbp stretch in the Cluster A region in the genomes of all serpentine-origin strains. The ~86 Kbp Cluster A region contains 32/33 Bonferroni-supported candidates, fully assorts with serpentine soil and contains nearly all of the candidates with annotations that suggest a role in nickel adaptation. However, there is evidence that two additional clusters of candidates supported at the 10% FDR level occur in subsets of the most nickel-tolerant strains, and 105 of the 202 candidate loci supported at the 10% FDR level are not clustered by our metric. The fact that serpentine-origin strains harbor at least one well-supported cluster of adaptive variants within a pan-genome otherwise mixed with non-serpentine strains by migration and recombination is consistent with at least one gene-specific sweep leading to the prevalence of candidates in Cluster A in nickel-adapted strains (Shapiro and Polz, 2014). Our data also suggest that multiple adaptive regions could act independently to confer nickel tolerance, which could further support additional gene-specific sweeps, though annotation-based support for these regions' involvement in nickel adaptation is less strong. Alternatively, historical genome-wide sweeps (clonal sweeps) could occur via strong selection in serpentine soils for adaptive variants in the absence of recombination. The resulting widespread clonal genotype could subsequently diversify by neutral processes, leaving locally adaptive regions under continued selection as remnants of ancient clonal divergence (Shapiro and Polz, 2014). These alternatives could be tested by assessing the relative pattern of polymorphism in candidate regions, and the level of synonymous divergence between habitats would shed light on which scenario is more likely (Shapiro and Polz, 2014).

Our findings support previous work showing that the accessory genome acts as a reservoir that harbors adaptive variation resulting from selection that impacts microbial populations at local ecological scales (Coleman and Chisholm, 2010; Doolittle and Zhaxybayeva, 2010; Polz *et al.*, 2013; Cordero and Polz, 2014). While many of the candidates we identify may be simply linked to adaptive variants, the functional annotation of numerous candidates suggests roles in nickel tolerance. Horizontal gene transfer, by which lineages can acquire genetic material from distantly related lineages, is one process structuring accessory genome content (Polz *et al.*, 2013). Typically, rates of horizontal gene transfer and recombination decline rapidly with sequence divergence between strains of bacteria (Polz *et al.*, 2013), negative epistatic interactions increase with divergence between source lineages (Polz *et al.*, 2013), and most horizontally transferred genes in rhizobia appear to be somewhat deleterious (Epstein *et al.*, 2014) and subject to strong purifying selection (Epstein *et al.*, 2014). However, our findings are consistent with a pattern by which selection maintains horizontally acquired genes shared with phylogenetically distant lineages. Strong, environmentally dependent selection on nickel tolerance could result in an excess of nickel-adaptive loci from distant lineages. Future closed genome sequencing would enable us to locate candidates within the replicon structure, as heavy metal tolerance in rhizobia can be conferred by plasmids (Lakzian *et al.*, 2002, 2007).

Our focal population shows adaptation to high-nickel serpentine soil, but does not show the pattern of reciprocal local adaptation found in the larger set of 292 *Mesorhizobium* strains (Porter and Rice, 2013). In the larger set, strains from high-nickel serpentine soil outperformed those from low-nickel non-serpentine soil in high-nickel media, and non-serpentine strains outperformed serpentine strains in low-nickel media. However, in the subset of strains in the focal population, non-serpentine ecotypes have no fitness advantage over serpentine ecotypes in the absence of nickel. This generalist population may have escaped the cost to metal tolerance observed in the larger, more phylogenetically diverse set of randomly selected environmental mesorhizobia (Porter and Rice, 2013), which could be due to migration alleviating the accumulation of environment-specific deleterious mutations (Kawecki, 1994) and could simultaneously be key to ongoing gene flow that prevents reproductive isolation across soil type boundaries (Shapiro and Polz, 2014). This lack of non-serpentine strain fitness advantage could have alternative explanations, such as a maladaptation load in non-serpentine strains due to recombination with serpentine strains. Future work could use a phylogentically controlled method to investigate how such trade-offs could result in varying patterns of gene flow to influence patterns of diversity (Gudelj *et al.*, 2010).

## Conclusion

We observe habitat specialization within an otherwise genomically cohesive population of wild mesorhizobia, rather than finding distinct, specialized bacterial lineages in contrasting soil types. Thus, heterogeneous environments can drive selection maintaining adaptive variants at a small fraction of the genome. Our work proposes candidate genes for nickel adaptation, several of which show homology to already-characterized genetic systems for nickel tolerance, which validates the general approach of ascribing habitat-assorting genomic variation to the processes of ecotypic adaptation.

## Figures and Tables

**Table 1:** Candidate genetic variants associated with nickel adaptation include variants functionally implicated in heavy metal tolerance (in bold)

*Variant*	*q-value*	*Ni phenotype effect*	*Frequency*		*Gene*	*Annotation*
			*S*	*N*		
SNP	0.024	0.22	1.00	0.59	GI:13476964	Succinoglycan transport protein exoP (non-synonymous, L/F)
Gene	5.13E-08	0.28	1.00	0.00	denovo003242	CBS domain-containing protein
	5.13E-08	0.28	1.00	0.00	denovo003246	Family transcriptional regulator
	5.13E-08	0.28	1.00	0.00	denovo003256	Membrane protein
	5.13E-08	0.28	1.00	0.00	denovo003257	Major facilitator transporter; H+ antiporter protein
	5.13E-08	0.28	1.00	0.00	denovo003258	**Cation diffusion facilitator family transporter**
	5.13E-08	0.28	1.00	0.00	denovo003265	**Opine dehydrogenase**
	5.13E-08	0.28	1.00	0.00	denovo003268	Peroxidase-related enzyme
	5.13E-08	0.28	1.00	0.00	denovo003270	ABC-type polar amino acid transport ATPase component
	5.13E-08	0.28	1.00	0.00	denovo003273	Amino acid ABC permease 3-tm his glu gln arg opine family
	8.17E-08	0.28	0.95	0.00	denovo003272	Amino acid ABC permease 3-tm his glu gln arg opine family
	2.14E-07	0.27	0.95	0.00	denovo003267	Flavoprotein involved in K+ transport
	4.32E-06	0.26	0.81	0.00	denovo003240	Recombinase
	1.38E-04	0.24	0.86	0.00	denovo003271	Periplasmic component of AA-type transporter signal transduction system
	1.58E-04	0.24	0.90	0.00	denovo003269	ABC-type polar amino acid transport ATPase component
	4.50E-04	0.22	0.95	0.41	denovo000014	**High-affinity nickel-transporter**
	5.85E-04	0.23	0.81	0.00	denovo003250	**Manganese and iron superoxide dismutase**
	7.96E-04	0.24	0.62	0.00	denovo003455	Aminophosphonate oxidoreductase; hydrolase; FAD dependent oxidoreductase
	9.69E-04	0.22	0.76	0.00	denovo003248	**Chromate ion transporter family**

The *q*-values indicate significance values for loci in an FDR-controlled association test on residual trait values corrected for weak genetic structure. Values remain significant after Bonferroni correction. Fifteen unannotated candidates are omitted. Positive Ni phenotype effect^a^ values indicate that a variant is associated with nickel tolerance rather than sensitivity. Frequency of the variant is given for strains from serpentine (S) and non-serpentine (N) soils. Annotations are based upon Blast2Go. Polymorphism type is parenthetically indicated for the SNP candidate.

a Ni phenotype effect is the mean growth (OD600) in Ni-enriched media of strains with the reference variant minus that of strains lacking the variant.

**Table 2:** Genes associated with nickel adaptation phenotypes in wild mesorhizobia that have supporting evidence in the literature

*Gene*	*Sig*	*Blast2GO*	*TrEMBL*	*Swiss-Prot*	*% AA ident.*	*% AA pos.*	*AA match length*	*Functional validation*
GI:13476964	Bonf. (SNP)	–	Succinoglycan transport protein exoP (*Mesorhizobium loti*)	Succinoglycan biosynthesis transport protein ExoP (*Ensifer meliloti*)	24	43	670	(Gad 1990; Harrison *et al.*, 2004; Jones *et al.*, 2008)
denovo003258	Bonf.	Cation diffusion facilitator family transporter	Probable Co/Zn/Cd cation efflux system protein (*Rhizobium etli*)	Probable cation efflux system protein Rv2025c encoding a deduced CDF-family metal exporter (*Mycobacterium tuberculosis*)	52	67	308	(Campbell *et al.*, 2007; Cubillas *et al.*, 2013)
denovo003265	Bonf.	Opine dehydrogenase	Opine dehydrogenase (*Mesorhizobium metallidurans*)	Opine dehydrogenase (*Arthrobacter* sp.)	28	48	348	(Dairi and Asano, 1995; Asano *et al.*, 1989)
denovo000014	Bonf.	High-affinity Ni transporter	High-affinity Ni transporter (*Sinorhizobium medicae*)	Ni/Co efflux system RcnA, encoding a membrane-bound polypeptide conferring increased Ni and Co resistance (*Escherichia coli*)	23	38	256	(Rodrigue *et al.*, 2005; Koch *et al.*, 2006; Iwig *et al.*, 2006)
denovo003250	Bonf.	Mn and Fe superoxide dismutase	Mn and Fe superoxide dismutase (*Burkholderia xenovorans*)	Superoxide dismutase [Fe] protein chrC (*Cupriavidus metallidurans*), part of a mechanism that destroys toxic superoxide anion radicals due to heavy metal exposure	64	75	194	(Juhnke *et al.*, 2002)
denovo003248	Bonf.	Cr ion transporter family	Cr ion transporter (CHR) family (*Ensifer meliloti*)	Cr transport protein chrA1 (*Cupriavidus metallidurans*)	25	45	362	(Nies *et al.*, 1990; Juhnke *et al.*, 2002)
denovo000013	10% FDR	Co–Zn–Cd resistance protein	Probable Co/Zn/Cd cation efflux system protein (*Rhizobium etli*)	Probable cation efflux system protein Rv2025c encoding a deduced CDF-family metal exporter (*Mycobacterium tuberculosis*)	69	82	79	(Campbell *et al.*, 2007; Cubillas *et al.*, 2013)
denovo003135	10% FDR	Oligopeptide dipeptide ABC ATPase subunit	Ni import ATP-binding protein NikD, putative (*Methylophaga thiooxydans*)	Oligopeptide transport ATP-binding protein YkfD (*Bacillus subtilis*)	51	68	297	(Han *et al.*, 2011)
denovo003284	10% FDR	Ni transporter permease	Ni transporter subunit membrane component of ABC superfamily (*Mesorhizobium metallidurans*)	Ni transport system permease protein NikC (*Escherichia coli*), translocates nickel through the bacterial inner membrane	37	60	249	(Navarro *et al.*, 1993)
denovo003460	10% FDR	Glutathione import ATP-binding protein; oligopeptide dipeptide ABC ATPase subunit; dipeptide ABC transporter ATP-binding protein	ABC-type dipeptide/oligopeptide/Ni transport system (*Methylophaga aminisulfidivorans*)	Putative peptide import ATP-binding protein BMEII0864 (*Brucella melitensis*)	47	63	311	(Han *et al.*, 2011)
GI:13475426	10% FDR	–	Ni–Co–Cd resistance protein (*Mesorhizobium loti*)	Ni–Co–Cd resistance protein NccN (*Alcaligenes xylosoxydans*); affects metal specificity, Ni resistance, may be involved in Ni transport	38	56	110	(Schmidt and Schlegel, 1994)

Annotations of candidate loci based upon: Blast2GO (for ‘*de novo*' genes not present in the reference), TrEMBL and Swiss-Prot databases. Summary statistics indicate homology between candidate loci and the closest match in Swiss-Prot. Sig, Significance: Bonf., Bonferroni-corrected candidate set; 10% FDR, 10% false discovery rate significance set; Ident., Identity; pos., positives; match length, number of overlapping amino acids between the consensus candidate sequence and the database sequence; Functional validation, citation supporting functional inference.
