# Supplementary material for: Association mapping reveals novel serpentine adaptation gene clusters in a population of symbiotic Mesorhizobium
Source: ISME J. 2016 Jul 15;11(1):248–62. doi: 10.1038/ismej.2016.88 (PMC5315480; doi:10.1038/ismej.2016.88)
Supplement: Supplementary Information [file ismej201688x1.docx]

**Supplemental Information 1. Supplemental Methods in a Word document.**

***Population pan-genomics***

**Strains.** The 48 Mesorhizobium strains studied here were previously isolated from field-collected legume root nodule tissue housing the symbiotic rhizobium bacteria by Porter & Rice (2012; Fig. 1). Briefly, randomly selected root systems including nodule tissue were collected from *Acmispon wrangelianus* host legumes, from both host patches on serpentine soil, a physiologically stressful natural soil enriched in toxic levels of nickel at the study sites (3.1 - 86.5 mg/kg Ni), as well as from spatially adjacent, benign non-serpentine soils containing lower levels of nickel (0.8 - 6.8 mg/kg Ni) (for full soil chemistry information, see Porter & Rice 2012), at each of three reserves within California (Fig. 1). One rhizobium isolate from one randomly selected nodule per plant was extracted using standard axenic culturing technique (Vincent, 1970) and preserved in 50% glycerol at -80°C. Sequence from the 16S ribosomal locus (Gaunt et al. 2001) was used to identify isolates as primarily lineages of the *Mesorhizobium* genus by blasting to the GenBank database. The isolated *Mesorhizobia* exhibited local adaptation to the presence or absence of nickel in their home soil, as demonstrated by growth assays under controlled laboratory conditions (Porter & Rice 2013).

We targeted a selection of 39 of these Mesorhizobium strains from a focal 16S subclade (99.8% sequence identity over 1340 bp of the 16S locus based on Sanger sequencing) of putatively cosmopolitan *Mesorhizobium* present in both soil types and present at all three reserves, as well as 9 strains representative of the diversity of other Mesorhizobium clades detected by Porter & Rice (2013). A maximum-likelihood based phylogenetic inference of strains’ 16S sequence was implemented in RAxML 7.2.6 using a GTRGAMMA model of molecular evolution (Stamatakis 2006) (Fig. 1A) . Focal clade strains were selected to include strains from geographically diverse sites, but were otherwise selected haphazardly. By analyzing published fitness data in the presence and absence of nickel in growth media for the focal 38 strains (Porter & Rice 2013) we verified this focal clade exhibits a strong pattern of adaptation to nickel. Strains from high-nickel serpentine and low-nickel non-serpentine soil differed in their response to the level of nickel in growth media (F_1,34_ = 78.4, P < 0.0001; 0 mM Ni enrichment vs. 1 mM Ni media enrichment). Strains collected from high nickel serpentine soil had higher fitness than strains from low nickel non-serpentine soil in high nickel growth media (F_1,34_ = 78.4, P < 0.0001; Fig. 1C). However, strains from high nickel serpentine and from low nickel non-serpentine soil had statistically indistinguishable fitness in low nickel media (Fig. 1).

**Genomic sequencing*.*** Isolates were grown in 10mL tryptone yeast broth cultures at 30°C shaking at 200 rpm in 15 mL centrifuge tubes until dense (3-5 days), then cells pelleted and frozen at -80°C. DNA extraction and genomic library construction followed the protocol of Dunham & Friesen (2013) with the following modifications. After 55°C for 30 min incubation, samples were transferred to a 96-well deep-well plate. Approximately 200 ul of each sample was transferred to another deep-well plate and Zymo lysis buffer was added. The remainder of the genomic DNA protocol followed manufacturer’s suggestions. Samples were quantified using Life Sciences (Invitrogen) QuBit fluorometer, and ~500 ng of DNA was used for genomic library construction. Libraries were sequenced in paired 76 bp format on an Illumina GAIIx at the University of Southern California.

**Assembly and annotation.** Draft genomes were assembled using the A5 pipeline (Tritt *et al*, 2012), using the May 18^th^ 2012 Linux x64 A5 version and run with default parameters. Genes were annotated using a two-stage procedure. First, draft genomes were searched for ‘reference’ genes homologous to 7,272 protein sequences found within the largest complete *Mesorhizobium loti* MAFF303099 genome (this strain is also known as *M. huakuii* bv *loti* (Zhang et al. 1996)). Genes homologous to those in the *M. loti* reference were considered present in an assembly if they were found by a BLASTX alignment that spanned longer than 80% of the length of an individual reference gene with E value less than 1E-20 and protein sequence identity greater than 50%.

Genes homologous to the reference could have been incorrectly identified as not present if the assembly resulted in sequence spanning less than 80% of a gene, for example, due to repetitive elements within a gene or if a gene was broken up into two different contigs. Due to the relatively liberal criteria for calling a gene present, this algorithm was unlikely to mistakenly identify genes homologous to the reference genome as not present and more likely to incorrectly identify such genes as present due to paralogous copies. Of the 7,272 gene sequences in *M. loti*, only 67 (0.9%) had a significant BLAST alignment to both themselves and another gene, which would indicate paralogy. No gene had a significant BLAST alignment to itself and to more than one other gene. This suggests that paralogy could drive a similarly small error rate in the reference gene-based annotation of genes in the wild *Mesorhizobium* strains we have sequenced.

Next, we identified genes present in the wild strains that were absent in the *M. loti* reference. Regions of the assembly that did not contain reference genes were aligned using BLASTX to the non-redundant GenBank CDS translations + PDB + SwissProt + PIR + PRF (nr) database available from GenBank using the same BLASTX parameters. Sequences with similarity to existing GenBank genes were aligned and clustered with other such sequences in other assemblies to identify “*de novo”* genes not present in the *M. loti* reference. To determine patterns of orthology among *de novo* genes, we used reciprocal BLAST hits to construct a directed graph, which was then split into subgraphs on weakly connected edges. We note that a given *de novo* gene may be present as multiple copies within a given draft genome, but simply treated them as present/absent. While it is impossible to know the rate of multicopy paralogs in our wild strains’ draft genomes, for our simulated data we found that ~15% of denovo genes mapped to more than one annotated gene per genome.

To obtain gene ontology annotations for both reference and *de novo* genes, we used Blast2GO v.2.3.5 (Conesa *et al.*, 2005), run with default parameters. The UniProt database of protein functional information was used to annotate *de novo* genes, with annotation performed separately for the sequence present in each strain. We then merged these annotations, retaining up to the top 5 highest-scoring homologs for each defined gene. We added annotations from the protein functional information databases, TrEMBL (automatically annotated) and Swiss-Prot (manually annotated and reviewed). TrEMBL generally yielded closer homologs to amino acid predictions for *de novo* genes but these specific homologs were generally not directly functionally validated, whereas Swiss-Prot generally yielded more distant homologs that were functionally validated via experimentation.

**Core and accessory genomes.** We defined ‘core’ genes to be those present in all strains for a given subset (38 for the focal clade, 48 for the entire Californian set, or 54 for all sequenced *Mesorhizobium* strains). Genes absent from one or more strain in each subset are referred to as ‘accessory’ genes relative to that subset. This delineation of core and accessory was calculated separately for each of the three subsets, depending on the analysis. In several of our analyses below, genes present in a single genome do not contain information regarding patterns of relatedness or biogeography and were thus excluded. For genes in the core genome across all 54 strains, we performed a multiple sequence alignment to identify single nucleotide polymorphisms (SNPs) using ClustalW. Only fully informative sites were used to call SNPs, i.e., gapless sites in the clustalw alignment of the genes taken from draft assemblies. This was done only on the full set of strains, which is conservative; SNPs in genes lacking from divergent *Mesorhizobium* strains will thus be lacking in our analysis.

For a single strain (NH2), 16S classification via Sanger sequencing places this strain within the focal clade, while the independent Illumina data places it outside the clade (Table S1, S2). This discrepancy could have resulted from human labeling error or non-isoclonal contamination in the cryogenically preserved stock for this strain. While this strain appeared consistent in genome size and characteristics with the other 47 wild Mesorhizobium strains, it was excluded from all downstream analysis.

**Simulating draft genomes to assess our pipelines.** To verify the efficacy of our two-step genome assembly and annotation algorithms in identifying accessory genes in a given genome, we simulated a set of 48 Illumina libraries from the six completely sequenced genomes available for congeneric *Mesorhizobium* lineages in Genbank (*M. alhagi, M. amorphae, M. australicum, M. ciceri, M. loti* and *M. opportunistum*).

To probabilistically calculate the core genome, we used the following model: If a gene has frequency p in the pangenome, we observe it in x out of n sampled genomes according to the Binomial distribution. We define a gene as belonging to the "core" genome if it is present at frequency p > p_c in the pangenome. The probability that a given gene is in the core genome, given that we observe it in x of n sampled genomes, is thus

*Pr(core | x,n) = Integral(binomial(x,n,p) on pc,1) / Integral(binomial(x,n,p) on 0,1)*

*= (Gamma[2 + n] (-Beta[pc, 1 + x, 1 + n - x] + (*

*Gamma[1 + n - x] Gamma[1 + x])/Gamma[2 + n]))/(*

*Gamma[1 + n - x] Gamma[1 + x])*

Multiplying this by the vector of genes observed in x strains yields the estimated number of core genes. Note that this assumes that the distribution of p in the population is uniform, which is not likely to be true. Evolutionary models of gene gain and loss (e.g., (Baumdicker *et al.*, 2012)) and additional data on the probabilities of not calling genes in draft genomes could be explored to generate a more sophisticated estimate of the core genome with experimental error.

Given the draft nature of our assembled genomes, some core genes could be incorrectly labeled as absent in a strain and therefore incorrectly considered accessory across the 48 wild strains. To determine the accuracy of our delineation of the core and accessory genomes, we performed additional simulations on each of the six completely sequenced strains from Genbank. For each completed genome, we simulated a set of 48 draft genomes assembled with sampled reads; 72 bp sequences were sampled randomly with replacement from each complete genome at a depth equal to each of the wild strain’s sequence dataset. We then analyzed them in using an algorithm analogous to that used on the reads from wild draft genomes. These simulations allowed us to estimate the likelihood of calling a gene as accessory due to the draft nature of our genome assemblies.

Our simulations *in silico* suggest the genome assembly algorithm produces a false discovery rate for delineating accessory genes (i.e., falsely identifying a gene as accessory when it is core) of less than 5% (Figure S1). Thus, despite the incomplete nature of the draft genomes assembled, we are confident in our delineation of ‘core’ and ‘accessory’ genes across our wild strains.

**Functional enrichment and molecular evolution.** GO enrichment tests were calculated separately for the core and accessory genomes with the R Bioconductor package ‘GOstats’, using GO.db_2.5.0 and the hypergeometric test with a nominal p-value of 0.05, using the conditional option to account for relationships between GO terms in the directed acyclic graph (Falcon & Gentleman, 2007). We compared rates of nonsynonymous (Ka) to synonymous (Ks) substitution in core and accessory genomes to examine whether the mean intensity of natural selection on protein coding genes differed between these portions of the pangenome. Analysis of molecular evolution was performed using KaKs_Calculator (Zhang *et al.*, 2006) on pairwise alignments between the *M. loti* reference gene and the sample genes generated using ClustalW. Only genes with a reference in *M. loti* were analysed due to the need to identify codon frame; we removed codons that contained insertions in the alignment. Therefore all core genes present in the focal clade and those accessory genes that occur in the reference *M. loti* (see visualization of genes not present in all 38 strains in Fig. 5C) were compared. KaKs_Calculator implements several candidate models in a maximum likelihood framework and uses Akaike’s information criterion to select the best model for the data and was run with default settings.

**Phylogenetics.** We explored relatedness among strains based on both allelic variation in their core genome and the pattern of gene presence/absence in their accessory genomes. We used a population-based perspective allowing horizontal gene transfer events between lineages that may otherwise evolve along asexual trajectories implemented in the NeighborNet algorithm (Huson, 1998). Calculations were performed in SplitsTree v4.12.8 (Huson and Bryant, 2006); 1000 bootstraps were performed for each network.

**Biogeography.** We assessed biogeographic structure among isolates from the three reserves and soil types in the core and accessory genome via two statistical tools. First, we analyzed the extent to which reserve and soil type explain variation in the core and accessory genome compartments in focal clade isolates using the non-parametric analysis of variance analog ‘adonis’ in the R package ‘vegan’ (Dixon, 2003). Adonis acts on distance matrices and we ran Euclidean, Bray-Curtis and Jaccard distance metrics with nearly identical results; those presented use Euclidean distances. As adonis is sensitive to the order of terms in the model, we conducted one-way analyses for soil and reserve separately. While estimates of the amount of genetic variance explained by these models may be inflated due to covariance between factors, this approach enables us to conservatively compare the amount of variance explained by these factors for the core and accessory genomes. To assess significance, we performed bootstrapping of strains (sampling with replacement) and calculated the mean and 95% range of each adonis variance component based on 100 bootstrapped data-sets equal in size to the original data.

To infer population structure and probabilistically assign individuals to populations, we ran STRUCTURE v2.3.2.1 (Pritchard et al., 2000) on each of 10 replicate SNP subsets, sampled every 25 polymorphic sites to reduce correlations, with K ranging from 1 to 8. Each run had a 10,000 step burnin with 20,000 post-burnin steps, sampled every 100 steps and repeated for 5 different seeds. We used Evanno’s method of delta_K = m(|L(K + 1) − 2 L(K) + L(K − 1)|)/s[L(K)] to determine the most likely value of K (Evanno et al. 2005). STRUCTURE was run once on the 48 strain-set, and 10 times on the 38 strain focal subclade based on 10 replicate SNP subsets. Table S3 presents these STRUCTURE assignments, including the mean assignment values across the 10 replicate runs for the focal clade.

***Adaptive variants***

**GWAS.** Rhizobia were previously phenotyped for nickel tolerance by comparing their growth (OD; optical density at 600 nm) in the presence (1 mM Ni) and absence (0 mM Ni) of nickel in tryptone yeast liquid media culture (Porter & Rice 2013). We conducted genome-wide association mapping of this growth phenotype in the presence and absence of nickel separately for (i) the accessory genome, i.e., the set of informative segregating genes, and (ii) SNPs in the core genome. Genes and alleles were considered informative if they were present or absent in at least two strains. The non-parametric Wilcoxon test was calculated for each individual variant and significance was assessed using Bonferroni correction as well as the more lenient false discovery rate (FDR) criterion

(Hochberg & Benjamini 1990). The Bonferroni threshold for association with the presence or absence of accessory genes is 1.04E-5 and the Bonferroni threshold for association with SNP variants is 2.36E-7. We conservatively regressed out STRUCTURE grouping effects for all traits by running a linear model of the trait values against the STRUCTURE assignment percentages (within the focal clade) and taking the residuals as the new, STRUCTURE-corrected phenotype values. This had very little effect on the number of Bonferroni-significant and FDR-significant genes and SNPs (Table S4 and S5); results in the main text focus on the STRUCTURE-corrected results. We note that the Bonferroni correction will be overly conservative, since the tests of genes linked on the same contigs with limited recombination are correlated rather than independent as assumed for this stringent control of the family-wise error.

To explore the potential biological functions determining growth in the presence of nickel, we examined the putative functions of genes (or genes containing SNPs) within these significance classes based on their inferred annotation. Additionally, for candidate loci with Blast2GO annotations that suggested a possible role in metal sensitivity, we calculated consensus sequences among the wild *Mesorhizobium* alleles within the focal clade and searched the TrEMBL and Swiss-Prot databases within the UniProtKB database to identify functionally validated published annotations with greatest homology to the candidates. Functional information in TrEMBL is automatically annotated and not reviewed, while the smaller set of functionally validated information in Swiss-Prot is manually annotated and reviewed.

Because most accessory genes are present in only one or two strains, and GWAS is most powerful for genetic variants that occur at intermediate frequencies, there could be additional genes associated with nickel tolerance we are unable to detect using this methodology.

**Accessory gene phylogenetic distance.** We tested whether candidate accessory genes are found in lineages more phylogenetically distant from the wild focal clade. Our general approach was the following: for each accessory gene in the focal clade strains, we identified the bacterial species bearing the closest BLASTX homolog for that gene, and then calculated the phylogenetic distance between this bacterial species and the focal clade strains at 16S. We then tested whether this phylogenetic distance differed for candidate vs. non-candidate accessory genes. Relatively even and slow rates of molecular evolution at the 16S locus across diverse taxa make it useful for comparing distantly related bacterial lineages. Thus, average nucleotide identities were calculated between the consensus 16S RNA sequence of the denovo strains and a representative sample of sequenced bacterial species, which include many of those that correspond to the non-reference genes identified to be orthologous to genes found in the denovo strains. Each of the bacterial 16S sequences in the ARB SSU Ref 115 non-redundant database were individually aligned to the consensus of our strains using Clustal Omega 1.2.0 with default parameters (CITES). Finally, the average nucleotide identities were calculated for each alignment by counting the number of mismatches in non-indel regions normalized by the length of the alignment with indels removed. For each gene, we selected the closest taxon based on the Genbank database and used that as our phylogenetic distance to the *Mesorhizobium* clade under investigation. When there were ties, we took the average phylogenetic distance. When the closest taxon was not in the 16S database we selected the closest relative based on the strain, e.g. "Rhizobium sp. BR816" -> "Rhizobium sp." We used a Wilcoxon signed-rank test to determine whether candidate genes for nickel tolerance were more distant than expected by chance, and a KS test to determine whether the distribution of phylogenetic distances differed between the set of candidate vs. non-candidate genes.

**REFERENCES**

Hochberg, Y. & Benjamini, Y., 1990. More Powerful Procedures for Multiple Significance Testing. Statistics in Medicine, 9(7), pp.811–818.

Porter, S.S. & Rice, K.J., 2013. Trade-offs, spatial heterogeneity, and the maintenance of microbial diversity. Evolution; international journal of organic evolution, 67(2), pp.599–608.

Stamatakis, A., 2006. RAxML-VI-HPC: maximum likelihood-based phylogenetic analyses with thousands of taxa and mixed models. Bioinformatics, 22(21), pp.2688–2690.

Zhang, X. et al., 1996. What does a bacterial genome sequence represent? Mis-assignment of MAFF 303099 to the genospecies Mesorhizobium loti. Microbiology, 148, pp.3330–3331.
