# Supplementary figures and images for: Association mapping reveals novel serpentine adaptation gene clusters in a population of symbiotic Mesorhizobium
Source: ISME J. 2016 Jul 15;11(1):248–62. doi: 10.1038/ismej.2016.88 (PMC5315480; doi:10.1038/ismej.2016.88)

**a**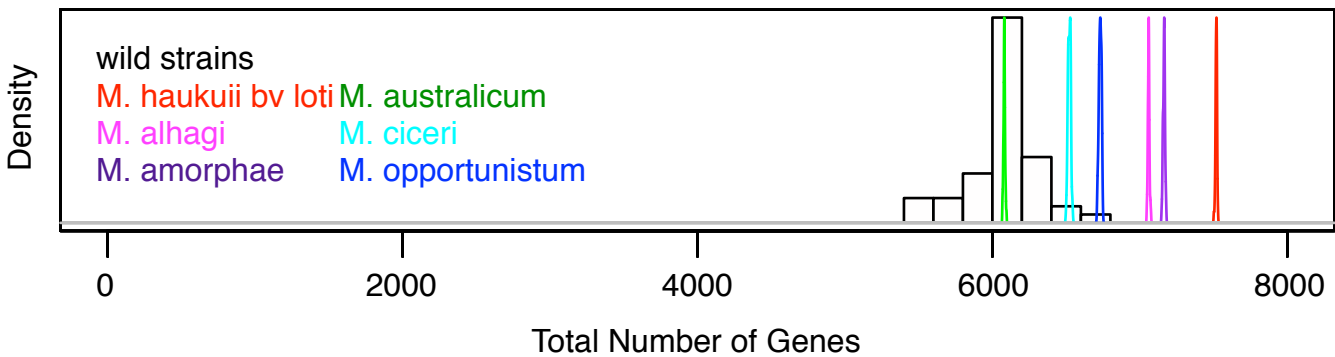**b**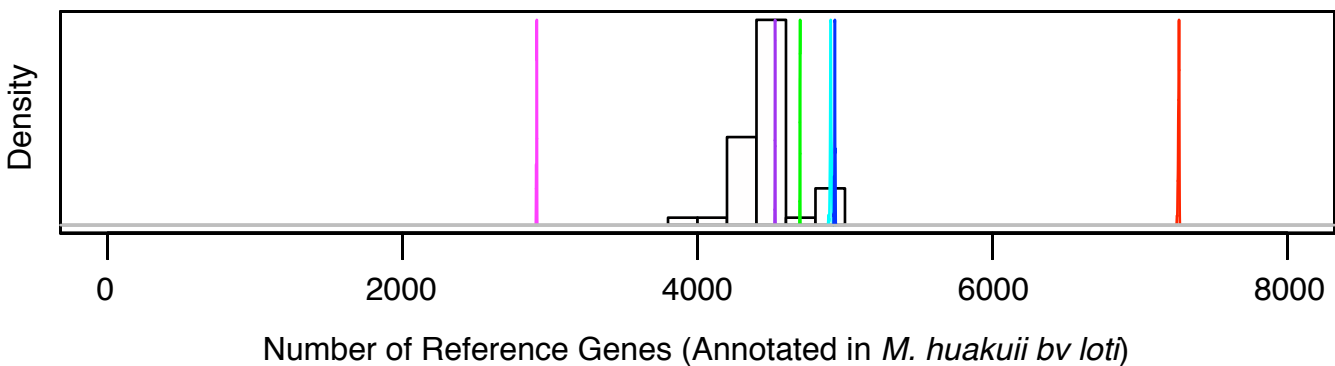**c**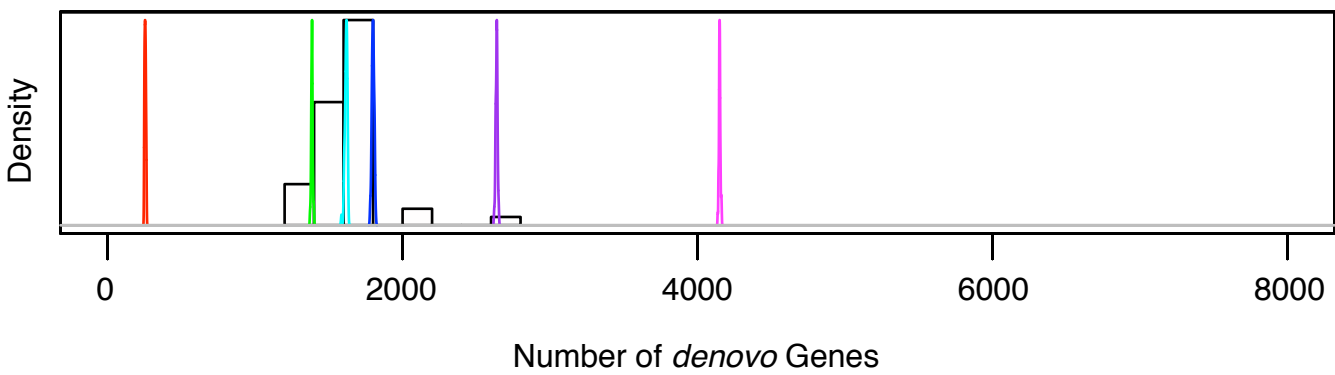

Supplement: Supplementary Figure 1 [file ismej201688x2.pdf]

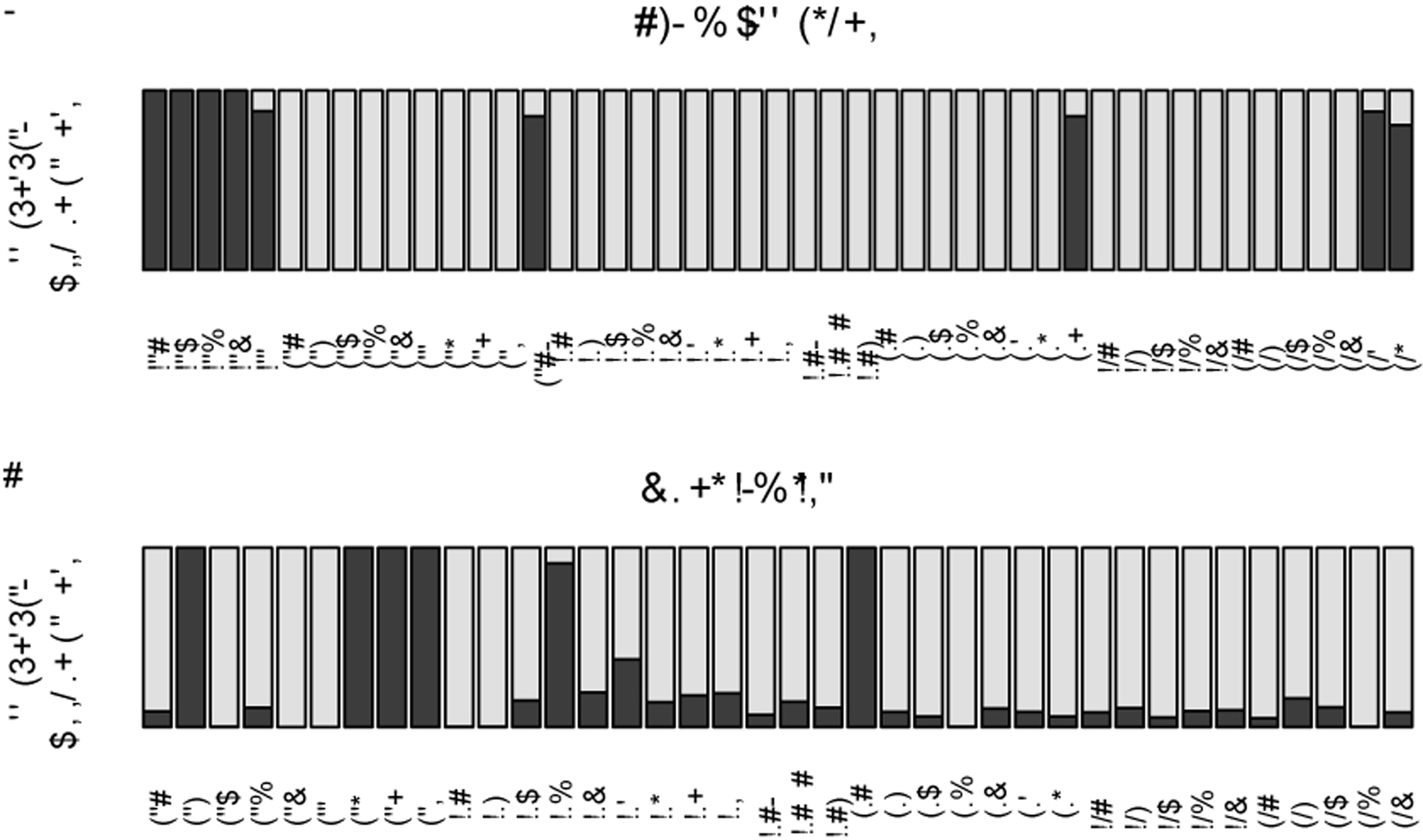

Supplement: Supplementary Figure 2 [file ismej201688x3.tif]

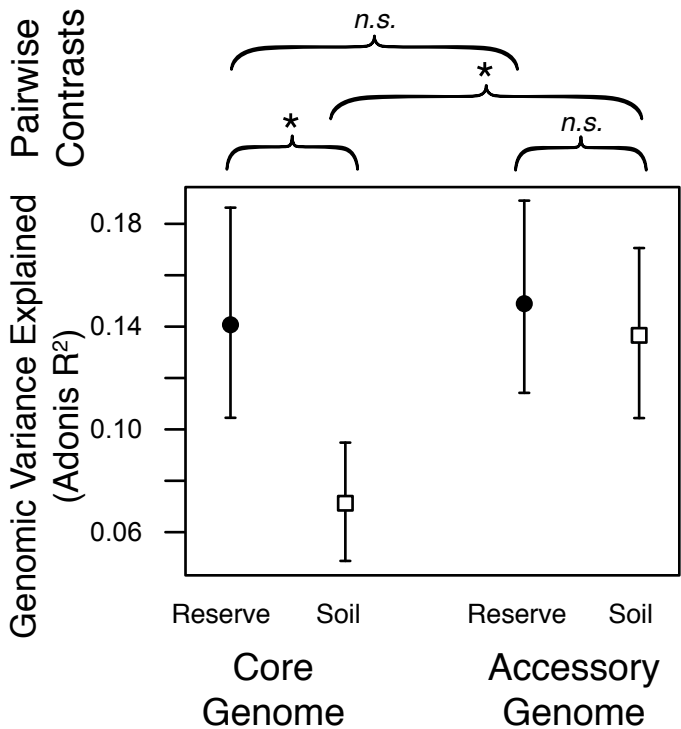

**FIGURE S3**

Supplement: Supplementary Figure 3 [file ismej201688x4.pdf]
